# Bacteriophages as New Human Viral Pathogens

**DOI:** 10.3390/microorganisms6020054

**Published:** 2018-06-16

**Authors:** George Tetz, Victor Tetz

**Affiliations:** 1 Human Microbiology Institute, 101 6th Street, New York, NY 10013, USA; vtetzv@yahoo.com; 2 Tetz Laboratories, 423W 127th Street, New York, NY 10027, USA

**Keywords:** microbiota disease, phagobiota, phagobiome, bacteriophage, microbiota, neurodegeneration, Alzheimer’s disease, Parkinson’s disease, PAMPs, autoimmune

## Abstract

The pathogenesis of numerous human multifaceted devastating diseases, including a variety of neurodegenerative and autoimmune diseases, is associated with alterations in the gut microbiota; however, the underlying mechanisms are not completely understood. Our recent human metagenome and phagobiota proteome analyses and studies in relevant animal models suggested that bacterial viruses might be implicated in the progression and maintenance of at least some pathologies, including those associated with protein misfolding. Here, for the first time, we propose the concept of bacteriophages as human pathogens. We suggest that bacterial viruses have different ways to directly and indirectly interact with eukaryotic cells and proteins, leading to human diseases. Furthermore, we suggest different causes of bacteriophages infection on the basis of the unique ways of interplay of phages, microbiota, and the human host. This concept opens a discussion of the role of bacteriophages as previously overlooked pathogenic factors and suggests that bacterial viruses have to be further explored as a diagnostic and treatment target for therapeutic intervention.

## 1. Introduction

The human gut microbiota is a highly diverse polymicrobial community composed of bacteria, archaea, fungi, and eukaryotic and prokaryotic viruses termed bacteriophages [1]. The microbiota performs functions that are key to human health and is essential for normal development and function of the gastrointestinal and immune systems, affecting all aspects of a human being including behavior and brain biochemistry [2,3,4,5]. The human intestine is colonized with microbiota during birth; however, some studies have indicated that that seeding occurs even before [6,7,8]. A notable feature of this community is its dynamic stability that is achieved through a variety of factors derived from the host macroorganism as well as from the microbiota [9,10]. Bacteriophages are the most abundant members of the gut microbiome—they outnumber intestinal bacteria over 10 folds—and they are one of the most important regulators of bacterial populations and microbiota stability [11].

Two main types of bacteriophages have been distinguished on the basis of their interaction with bacterial cells. The first type are lytic or so-called lytic phages that replicate inside the bacterial host cells and kill the bacteria to release progeny particles at the end of their cycle [12,13]. Of note, this lytic potential of phages gave rise to the application of such phages for the treatment of bacterial infections [14,15]. The second type, named lysogenic or temperate phages, can reproduce using both the lytic and the lysogenic cycles [16]. During the lysogenic cycle, the bacteriophage introduces its genome into the host cell’s DNA to replicate passively with the host, without producing virions; however, these lysogenic phages can also switch to the lytic state upon induction, killing their host by progeny release and thus reducing bacterial abundance [17,18]. 

Although alterations of the gut microbiota have been suggested to be implicated as an important trigger and worsening factor of various multifaceted human diseases, including neurodegenerative pathologies and certain cancers, until recently, very few studies looked into the role of bacteriophages in human health [19,20,21]. One of the best described disease-specific alterations in the gut phagobiome is associated with inflammatory bowel disease, where phages have pathogenic effects by inducing dysbiosis and microbiota disease, and attempts have been made to use phage transplants as a therapeutic approach to re-establish a healthy microbiota [22,23]. Altered bacteriophages along with dysbiosis have been also observed in subjects with periodontal disease, suggesting that viruses may be implicated in oral health as well [24,25].

Currently, causes of human disease are generally thought to be explainable by the genetic variation theory, the Germ Theory, or by exposure to negative factors, such as carcinogens, in the outer environment and/or through diet and lifestyle [26,27,28]. Specific genetic variations may be a result of genetic predisposition inherited from parents or due to the accumulation of mutations during life caused by the outer environment [29,30]. These genetic changes contribute to the development of a disease, but do not directly cause it. Some people with a predisposing genetic variation will never get the disease while others will, even within the same family [31,32]. The Germ Theory of Disease states that many human diseases are caused by microbes. The theory was originally introduced by Louis Pasteur and was later extended by Robert Koch, and initially attributed to bacteria [26]. Today, the term “germ” refers not just to bacteria but also to fungi, protozoans, eukaryotic viruses, etc. [33,34]. However, bacterial viruses were never suggested as human pathogens, as they were believed to affect only microorganisms [34].

**Figure 1:**
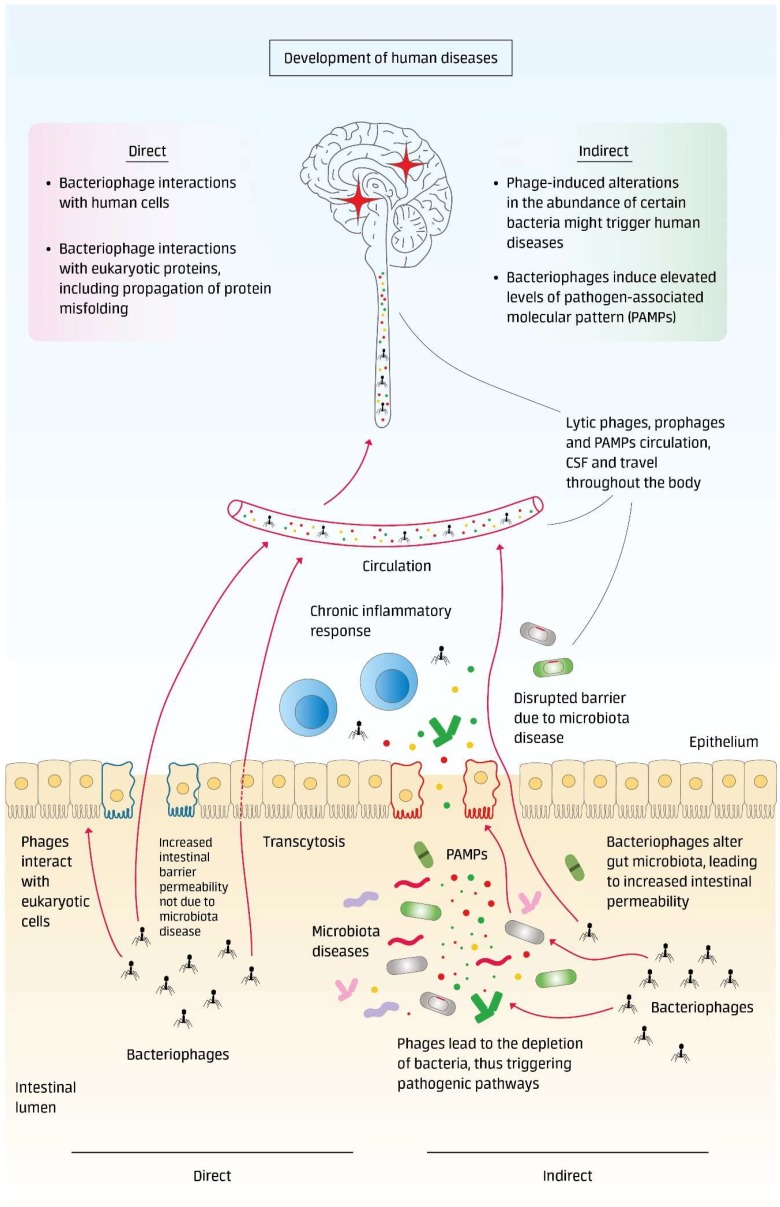
Bacteriophage concept of human diseases. Direct interaction of phages with the host macroorganism (eukaryotic cells and proteins). Indirect interaction with the host macroorganism by causing harmful microbiota alterations and microbiota-related diseases.

Here, we introduce the concept of bacteriophages as human pathogens. Recent discoveries made by our and other scientific research teams provide evidence that bacteriophages are overlooked human pathogens, implied in the triggering, and worsening of a number of human diseases [19,20,35]. We suggest a bacteriophage concept of human diseases consisting of the following features:

- Direct interaction of phages with the host macroorganism (eukaryotic cells and proteins);
- Indirect interaction with the host macroorganism by causing harmful microbiota alterations and microbiota-related diseases (Figure 1).

### 1.1. Phages as Human Pathogens: Direct Actions of Phages

#### 1.1.1. Model 1. Bacteriophage Interactions with Human Cells

Phages can cross barriers and access different parts of the human body in multiple ways. Recent studies have shown for the first time that free bacteriophages can interact directly with eukaryotic cells, allowing their transcytosis across confluent epithelial cell layers, therefore modeling phage-eukaryotic interactions in the gastro-intestinal tract [36]. Although the molecular mechanisms of phage-eukaryote interactions during translocation remain elusive, the finding raises important questions about the previously unknown intracellular effects of phages within eukaryotic cells [37,38].

In recent experiments, after oral administration of different bacteriophages in mammalian animal models, we detected the phages in the blood, as well as increased intestinal permeability, regardless of whether bacterial hosts existed in the gut microbiota. Following oral administration, we recovered *Escherichia*, *Staphylococcus*, *Klebsiella*, *Bacillus*, and *Paenibacillus* phages at high concentrations (5–10 log10 PFU/mL) from the blood of the animals (unpublished data). Notably, the strictly virulent *Listeria* phage P100, which is widely used in the processing of poultry to control *Listeria*, was also detected in the circulation when added to the drinking water [39,40]. In agreement with previous reports of the detection of phages in human biological fluids, we detected the dissemination of all bacteriophages tested to the spleen, which is responsible for bacteriophage clearance from the circulation [41,42,43,44,45]. 

Along with the discovery of phagemia due to increased intestinal barrier permeability, we have evaluated the presence of phages in human cerebrospinal fluid (CSF). CSF is a class of transcellular fluids that originates from the plasma and is suggested to be sterile under normal conditions [46]. Originally, the appearance of bacteria and viruses in the CSF was associated with a number of life-threatening infectious diseases, particularly, various forms of bacterial and viral meningitis [47,48,49]. However, recent studies associated certain viruses and bacteria in the CSF with the development of neurodegenerative diseases. For example, HHV-1 and chlamydia are strongly associated with Alzheimer’s disease (AD), and have been suggested to play a role in the formation of amyloid beta plaques, while the presence of HHV-6 in the CSF has been associated with multiple sclerosis [50,51,52]. The pathogenic mechanisms of bacterial and viral presence in the CSF in the development of neurodegenerative diseases are multifaceted and include the development of an autoimmune cascade and an altered inflammatory response [53]. 

We first observed the presence of bacteriophages in the CSF of subjects with neurodegenerative pathologies, opening the discussion on their potential link. We have detected the presence of *Shigella* phage SfIV and *Staphylococcus* phage StB2 in the CSF of patients with multiple sclerosis (MS), compared with 15 control patients with other neurological diseases (unpublished data). Although we have not studied the role of these phages in this disease, their circulation in the CSF of MS patients points to the possible pathological role, as in the case of the presence of eukaryotic viruses in the CSF of patients with other neurodegenerative diseases. Interestingly, patients with MS are characterized by increased intestinal permeability and a disrupted blood-brain barrier, suggesting a possible phage transport route from the gut to the CSF and the brain [54,55]. Within this framework, we can speculate that, as there are multiple conditions that are characterized by leaky gut, resulting phagemia can lead to the entrance of phages into the CSF much more often than currently thought, and the link between phages and neurodegenerative conditions deserves in-depth study. The circulation of phages in the CSF has been overlooked likely because many metagenomic studies of human biological fluids are based on 16S RNA gene sequencing, which allows the identification of bacterial species but not bacteriophages, which require shotgun sequencing [56]. Phages have been shown to pass through the placental barrier, which expands the concept of phage transfer beyond the gut [37]. All these data point out that phages can be transmitted to different organs, including the brain, via the circulation. Therefore, based on the discovery by Nguyen et al. of phage-eukaryote interactions in the gut lining, we believe that such an interaction can occur in any other tissue the phages reach, including the nervous system, meaning that additional research is required to elucidate the role of phages circulating in human biological fluids in health and disease [36]. 

#### 1.1.2. Bacteriophage Interactions with Eukaryotic Proteins, Including Propagation of Protein Misfolding

We were the first to identify a variety of prion-like domains that have a strong capability to become prions in a variety of bacteriophages, including those associated with the human microbiota [57]. We speculated that the prion domains in bacteriophage proteins may be involved in cross-kingdom interactions with eukaryote proteins and in protein misfolding in humans. Protein misfolding and the formation of prions has become the leading theory for the development of different multifaceted diseases, including autoimmune and neurodegenerative disorders [58,59]. Although the key molecular and cellular events underlying the development of AD, Parkinson’s disease (PD), amyotrophic lateral sclerosis, and other disorders are clearly divergent, they all have the common features of protein misfolding and formation of aggregates that possess cellular toxicity, leading to neuronal alterations and death due to the formation of prion proteins [60,61]. Prions are characterized by self-propagation, conformational switch capacity, formation of form β-sheet like motifs, and creation of new, misfolded proteins [60,61,62]. The mechanisms underlying protein misfolding and prion formation remain elusive. In favor of our hypothesis that phage interactions with human proteins may be associated their misfolding, Chen et al. recently reported that *Caenorhabditis elegans* fed prion-producing *Escherichia coli* showed enhanced prion aggregation in the brain, suggesting cross-kingdom bacteriophage interaction [63]. It can be assumed that a similar process may be observed following the introduction of bacteriophages enriched with prion-like domains, and that bacteriophages contribute to such processes observed in nature. Additional evidence for the role of phages in mammalian protein misfolding can be found in a recent study describing the use of bacteriophage M13 to reverse the formation of plaques derived from amyloid-like structures in the brain [64]. Notably, in our previous analysis of prion-like domain distribution among phagobiota, M13 phages were found to possess multiple prion-like domains within the attachment protein G3P [57]. Moreover, the discovery of phages in human biological fluids opens the discussion on the role of phage-induced protein misfolding in the blood circulation and CSF.

### 1.2. Phages as Human Pathogens: Indirect Interactions through Microbiota Alterations and Microbiota Diseases

#### 1.2.1. Phage-Induced Alterations in the Abundances of Certain Bacteria Might Trigger Human Diseases

As mentioned earlier, bacteriophages can interact with bacteria through lytic infection or lysogenic infection, both of which can lead to lysis of bacterial host cells, significantly altering certain bacterial populations and thereby indirectly contributing to the shift from health to disease in mammals [65,66,67]. Under this model, phages cause microbiota alterations, which in turn are implicated as a trigger or worsening factor of different multifaceted diseases, such as various neurodegenerative pathologies and certain types of cancers characterized by the implication of microbiota and autoimmunity. 

We recently discovered the effect of lytic bacteriophages on the microbiota of patients with PD that might contribute to the onset of this pathology [68]. We showed that the abundance of lytic *Lactococcus* phages was higher in patients with PD than in healthy individuals and was associated with a 10-fold reduction in neurotransmitter-producing *Lactococcus* bacteria, which suggested an association with and the possible role of phages in neurodegeneration. *Lactococcus* bacteria are important producers of neurochemicals in the enteric nervous system and are regulators of gut permeability. Thus, their depletion due to high numbers of strictly lytic phages in PD patients might be associated with gut-originating PD pathogenesis, a model that is based on the idea that alterations in the gut microbiome and bacteria-originated neurotransmitters play a role in triggering α-synuclein misfolding in the enteric nervous system and in turn spread through the vagus nerve to the central nervous system [69,70,71]. Moreover, our results indicated that the decrease in lactococci in the PD group was due to the appearance of strictly lytic, virulent lactococcal phages belonging to the c2-like and 936 groups that are frequently isolated from dairy products, opening a discussion on the role of environmental phages and phagobiota composition in health and disease [62]. 

Interestingly, *Lactococcus* spp. are known to possess abortive infection (Abi) systems that function to block phage multiplication [72]. Abi leads to premature bacterial death following phage infection, thus decreasing the number of progeny phage particles and limiting their spread to other bacteria within the population. This system can be overcome only by phages carrying certain mutations. In the case of PD patients, we suggest that most likely, the identified lactococcal phages have overcome these antiphage systems, being so called Abi-escape phage mutants, and point out the particular role they can play in human health [68,73].

In another way of indirect interaction, phages can affect human hosts through the induction of increased intestinal permeability. We were the first to show that bacteriophages could induce leaky gut and an impaired intestinal barrier with an elevated lactulose/mannitol ratio, due to a cascade of microbiota alterations finally resulting reduced *Lactobacillus* spp. and *Feacalibacterium* spp., which are important regulators of the intestinal barrier [19,20]. The phage-induced intestinal permeability was accompanied with an increase in plasma endotoxin concentrations and elevation of inflammation-related cytokines, reflecting chronic inflammation. The fact that phages have been previously overlooked as a cause of increased intestinal permeability is notable as leaky gut is a well-known trigger of various poly-etiological diseases associated with chronic inflammation and underlies the development of various multifaceted diseases, such as AD, PD, amyotrophic lateral sclerosis, chronic fatigue syndrome, diabetes, autism, and certain cancers [74,75]. Moreover, phage-induced altered gut barrier results in phagemia and the circulation of phages in the CSF contribute to phage interplay with human cells and proteins that normally are not exposed to phages.

#### 1.2.2. Bacteriophages Induce Elevated Levels of Pathogen-Associated Molecular Pattern (PAMPs)

In our latest work, we showed that the oral administration of bacteriophages results in increased levels of plasma cell-free bacterial DNA derived from gut bacteria (unpublished data). We showed in vitro that bacterial phage-induced lysis leads to the release of bacterial DNA from microbial biofilms, which are known to be the main form of bacterial existence in the human gut [76]. Based on these findings, we suggested that similar processes might occur in vivo, and lysis of bacterial populations under lytic phages or induction of lysogenic infection might be associated with the entry of released bacterial DNA into the blood stream. In follow-up in-vivo experiments, we detected elevated levels of cell-free DNA in the systemic circulation as early as within 24 h following bacteriophage oral administration, using qPCR to prove that the DNA was derived from bacteria [77]. Of note, circulating bacterial DNA is known as an important source of PAMPs thus, this opens the possibility of the implication of phages in altered immune responses due to elevated PAMP levels [78,79]. Moreover, one can suggest that other PAMPs, such as LPS, peptidoglycan, and bacterial amyloid can enter the blood circulation particularly in subjects with substantially increased intestinal permeability; however, this requires further study [80]. Notably, given that increased gut permeability facilitates the translocation of PAMPs into the circulation, under certain conditions, particular bacteriophages might have a dual role, inducing gut leakiness on the one hand and causing bacterial lysis, leading to elevated serum PAMP levels, on the other hand [81].

Moreover, phages, carrying DNA and RNA, are in themselves an important source of PAMPs [82]. Bacteriophage-derived nucleic acids are recognized by multiple TLRs, including TLR3, TLR7, TLR8, and TLR9, which induce the production of type I IFN, known to be implicated in the etiology of different autoimmune pathologies, including type 1 diabetes and systemic lupus erythematosus [83,84,85,86]. As we have shown, phages have multiple direct and indirect ways to affect macroorganisms, and the study of their implication in human diseases requires inventive approaches that differ from those used to study bacterial and eukaryotic viral infections. Compared to the Germ Theory of Disease, the bacteriophages concept of human diseases is more complex. It implies different direct as well as indirect effects that are realized thought the presence of bacterial hosts within the human microbiota, thus rendering an additional level of complexity.

We believe that there are certain predispositions in macroorganisms that render them more susceptible to the effects of bacteriophages, including (a) genetic variations in macroorganisms with predisposition to certain human diseases enabling the realization of phages’ pathogenic potential, (b) genetic variations in macroorganisms enabling patterns of microbiota, thus providing development of particular conditions for bacteriophages, that in turn can trigger host pathologies, (с) microbiota particularities not associated with genetic variations of humans, allowing a negative effect of phages on the macroorganism, and (d) a combination thereof.

It is noteworthy that there are causes of bacteriophage infections that reflect their infection routes in humans (Table 1).

In primary bacteriophage infection, humans are directly infected by free lytic phages or by prophages that become free virions following lysogenic induction after entry into the gut [12]. Notably, phages that infect humans can originate from different sources in the outer environment, as they are generally resistant to a variety of unfavorable conditions [88]. Moreover, prophages of spore-forming bacteria are even more protected in the endospores, rendering them resistant to nutrient and water deprivation, antimicrobial agents, and the host immune system [89,90]. Most likely, phages can spread in an epidemic-like manner in urban areas, for example, via water and widely used dairy products [91,92,93]. Another way of phage entry into the human microbiota is through the consumption of foods industrially processed with phages to control foodborne bacteria [39,49]. In addition, dramatic shifts in the host phagobiota have been observed following fecal microbiome transplantation and led to a profound and long-lasting alteration of phage contents in the recipient’s microbiota [94]. Of particular interest are hospital-acquired free bacteriophages and prophages. We outline them as nosocomial-derived bacteria that have an elevated mutation rate that also affects prophage DNA, endowing progeny phages within new properties, including those associated with the ability to overcome bacterial defense systems [95].

Overall, the fact that phage transmission occurs from the outer environment as well as between humans, outlines the possible contagiousness of the diseases associated with them [19]. It can be speculated certain phages in humans with genetic or microbiota-based predisposition can be associated with the development of different poly-etiological pathologies mentioned above, including neurodegenerative ones. 

Other causes of bacteriophage infections encompass bacterial viruses that are present in humans and are harmless under normal conditions, but can become pathogenic under certain circumstances. This can be due to an increased translocation (for example due to impaired intestinal permeability caused by any underlining condition) of phages to biological fluids, leading to profound direct phage interactions that normally do not occur. Another cause is an increase in the numbers of prophages or free lytic phages as a result of a shift in the microbiota [88,96]. Furthermore, certain mutations in prophage genes that arise within the human microbiota can lead to the formation of viruses able to overcome bacterial defense systems, for example, the so-called Abi-escape phage mutants [73]. Finally, we suggest that an individual can be more or less susceptible to bacteriophage infection under certain circumstances. Notably, the alterations in susceptibility are realized on the levels of macroorganism or microbiota sensitivity to phages, which can be altered by a variety of different factors. 

Although the pathological roles of phages in human diseases are just being discovered, further determining the roles of phages in triggering and maintaining human diseases is very promising and might lead to novel interventional approaches in different areas of medicine.

## 2. Discussion

The pathogenesis of neurodegenerative and autoimmune diseases is believed to involve a complex interaction between genetic predisposition and environmental factors, including microorganisms and microbiota [97]. Although alterations of the microbiota are known to be crucial for the development of such pathologies, previous studies focused on the roles of bacteria, fungi, and eukaryotic viruses, while bacteriophages were not considered to play a role in human health [20,21,22,23,24,25]. Here, for the first time, we presented the concept of bacteriophages as human pathogens, implying phages as previously overlooked factors that might be associated with human diseases. This concept adds phages and phagobiota to the growing list of factors associated with human health, and moreover suggests that bacteriophages and the alteration of their abundances may be a target for therapeutic intervention. Substantial additional experimental work is required to distinguish which bacteriophages contribute to the development of human diseases and to evaluate the roles of human’s genetic and microbiota susceptibilities therein. As a result, phages may become a novel frontier for disease diagnostics, treatment, and prevention.

## Figures and Tables

**Table 1:** Causes of bacteriophage infections.

Main Causes of Bacteriophages Infections	Comments
Infection by lytic phages	Primary bacteriophage infection due to environmental bacterial viruses
Infection by bacteria carrying prophages in their DNA	Primary infection by environmental bacteria harboring prophages
Induction of prophages	Can be due to a variety of different internal and external triggers, leading to progeny release, an increase in the number of free phages leading to significant microbiota alterations [87]
Increased translocation of phages to biological fluids	Can be due to increased intestinal and blood-brain barrier permeability [43,81]
Alteration of the microbiota	Alteration of microbiota composition (following emergence of new bacteria, antibiotics treatment, diet, etc.) leading to a shift in numbers of prophages or lytic phages
Mutations in prophages leading to Abi-escape phage mutants	Phages able to overcome bacterial defense systems [72,73]
Changes in macroorganism sensitivity to direct and indirect bacteriophage effects	Human sensitivity to bacteriophage infection can be altered through a variety of internal factors or factors associated with the microbiota
